# Interleukin-32θ inhibits tumor-promoting effects of macrophage-secreted CCL18 in breast cancer

**DOI:** 10.1186/s12964-019-0374-y

**Published:** 2019-05-24

**Authors:** Thu-Huyen Pham, Yesol Bak, Taeho Kwon, Sae-Bom Kwon, Jae-Wook Oh, Jong-Hyung Park, Yang-Kyu Choi, Jin Tae Hong, Do-Young Yoon

**Affiliations:** 1 Department of Bioscience and Biotechnology, Konkuk University, 120 Neungdong-ro, Jayang-dong, Gwangjin-gu, Seoul, 05029 Republic of Korea; 2 Primate Resource Center, Division of Bioinfrastructure, Korea Research Institute of Bioscience and Biotechnology (KRIBB), Jeongeup, Jeollabuk-do Republic of Korea; 3 Department of Stem Cell and Regenerative Biotechnology, Konkuk University, Seoul, Republic of Korea; 4 Department of Laboratory Animal Medicine, College of Veterinary Medicine, Konkuk University, Seoul, Republic of Korea; 5 College of Pharmacy and Medical Research Center, Chungbuk National University, Osongsaengmyeong 1-ro, Osong-eup, Heungdeok-gu, Cheongju, Chungbuk 28160 Republic of Korea

**Keywords:** IL-32θ, Macrophage, Breast cancer metastasis, CCL18, PKCδ, STAT3

## Abstract

**Background:**

Tumor-associated macrophages can promote breast cancer metastasis by secreting cytokines and growth factors. Interleukin (IL)-32θ, a newly identified IL-32 isoform, was previously shown to down-regulate various proinflammatory factors of macrophages. Here, we report the presence of IL-32θ in breast cancer tissues and evaluate its effects on macrophage-regulated breast cancer metastasis.

**Methods:**

RT-qPCR was used to analyze the mRNA expression of IL-32θ, Chemokine (C-C motif) ligand 18 (CCL18) in breast cancer tissues. In vitro cell-based experiments using IL-32θ-expressing MDA-MB-231 cells were conducted to examine the effects of IL-32θ on metastasis and its molecular signaling. In vivo xenograft, immunohistochemistry, and optical imaging models were generated to support in vitro and clinical findings.

**Results:**

The clinical data displayed opposite expression patterns of CCL18 and IL-32θ mRNA in macrophage-infiltrated breast tumor tissues compared with those in the other tissues tested. In MDA-MB-231 cells, IL-32θ overexpression attenuated migration, invasion, tumor-promoting factors, and increased epithelial markers levels upon treatment with conditioned media from THP-1-derived macrophages. Additionally, IL-32θ expression in a xenograft model led to a remarkable decrease in tumor size and macrophage-stimulated tumor promotion. This inhibition was mediated through a direct interaction with protein kinase C-δ (PKCδ), subsequently eliminating the downstream factors STAT3 and NF-κB. Blocking CCL18 during co-culture of macrophages and breast cancer cells reduced the levels of breast cancer progression-related factors and PKCδ downstream signaling suggesting CCL18 as the main macrophage-secreted factors triggering the signaling pathway inhibited by IL-32θ.

**Conclusions:**

Our findings demonstrate a novel role of IL-32θ as an intracellular modulator to suppress macrophage-promoted breast cancer progression by targeting CCL18-dependent signaling.

**Electronic supplementary material:**

The online version of this article (10.1186/s12964-019-0374-y) contains supplementary material, which is available to authorized users.

## Background

Breast cancer is the most common cancer in females worldwide, and is also the leading cause of cancer-related death in the majority of countries [1]. Tumor progression is the process by which tumor cells acquire more aggressive and malignant characteristics, allowing them to invade microenvironments and subsequently migrate to distant organs [2, 3]. In this process, epithelial-mesenchymal transition (EMT) is one of the key events that allows tumor cells to switch to mesenchymal phenotypes to facilitate their migration, invasion, and metastasis [4]. This tumor metastasis and acquired resistance to tumor therapy is a result of the interaction between cancer cells and the tumor microenvironment, leading to the secretion of various factors that target cancer cells and manipulate their promotion [5–7]. Therefore, inhibition of these interactions can serve as a therapeutic approach in cancer.

Macrophages are the most abundant immune cells in the tumor microenvironment, which can occupy up to 50% of the entire tumor mass [8], and have been associated with poor outcomes in various carcinomas [9]. Macrophages can be classified into M1 and M2 macrophages, which polarize into the respective forms in response to an environmental change. M2 macrophages facilitate angiogenesis, tissue remodeling [10], and promote breast cancer progression by secreting angiogenic factors and breast tumor mitogens [11]. Tumor-associated macrophages (TAMs) are a type of M2 macrophages, and breast cancer TAMs display an alternative phenotype that promotes tumor invasion and metastasis [12]. Further, cancer cells can educate macrophages to enhance tumor development and metastasis [6]. Numerous studies have determined the relationship between breast cancer and macrophages, and cancer therapies targeting both breast cancer cells and macrophages are of great interest given their potential in the clinical setting.

Interleukin (IL)-32 was first reported as natural killer transcript 4 located on human chromosome 16p13.3 [13]. IL-32 has various isoforms due to alternative splicing [14], although the role of each isoform in disease remains controversial [15]. Among the IL-32 isoforms, our group discovered both IL-32θ and IL-32 small fragment [14] and reported the functions of IL-32θ in inflammation and cancer [16–18]. In the present study, we aimed to investigate the role of IL-32θ in the breast cancer microenvironment and to determine whether IL-32θ could suppress macrophage-induced breast cancer progression, and to explore the underlying molecular mechanisms.

## Materials and methods

### mRNA extraction from breast cancer tissue

The biospecimens including breast tissues (*n* = 90) and sera (*n* = 55), and the characteristic information of breast cancer patients used in this study were provided by the Biobank of Chonnam National University Hwasun Hospital (Hwasun-gun, Korea) and Korea University Guro Hospital (Seoul, Korea). RNA was extracted from frozen tissues using a homogenizer and TRI Reagent® (Ambion, Austin, TX), and then cDNA was synthesized using the M-MuLV reverse transcriptase (New England Biolabs, Beverly, MA) according to manufacturer’s instructions.

### Cell culture and treatment

The MDA-MB-231 cell line (ATCC® HTB-26™, Manassas, VA) was cultured in DMEM (Hyclone Laboratories, Logan, UT). The human monocytic cell line THP-1 (Korean Cell Line Bank, Seoul, Korea, KCLB-40202) was cultured in RPMI-1640 (HyClone). Both mediums were supplemented with 10% heat-inactivated fetal bovine serum (MilliporeSigma, Burlington, MA), 100 units/mL penicillin, and 100 μg/mL streptomycin at 37 °C/5% CO_2_. To generate the conditioned media (CM), THP-1 cells were stimulated with 100 nM phorbol ester (PMA) (MilliporeSigma) for 48 h, the non-attached cells were washed with phosphate buffered saline (PBS) followed by addition of fresh culture media, and these cells were then incubated for another 24 h. The CM was collected and centrifuged to remove the remaining cells.

### Generation of the IL-32θ-overexpressing cell line

We transfected MDA-MB-231 cells with the pcDNA3.1 (+)-6 × Myc-IL-32θ vector or pcDNA3.1 (+)-6 × Myc-empty vector, as described previously [19] and refer as MDA-MB-231-IL-32θ and MDA-MB-231-EV cells, respectively. In brief, the cells were seeded into 6-well plates (1 × 10^5^ cells/well) and transfected with 3 μg of vector using Lipofectamine® 2000 (Invitrogen, Carlsbad, CA). Afterwards, the cells were selected using medium containing 700 μg/ml G-418 (Duchefa Biochemie BV, Haarlem, The Netherlands) for two weeks. G-418-resistant colonies were then pooled and expanded.

### Migration and invasion assays

For the migration assay, cells (5 × 10^5^ cells/mL) were seeded onto the upper chambers of 24-well transwell plates (Corning Inc., Corning, NY) in serum-free DMEM. For the invasion assay, each transwell chamber was coated with 30 μl of Matrigel (Corning) before breast cancer cells were added to the top chamber. The lower chamber contained 500 μl macrophage-derived CM. Cells migrated or invaded for 24 h at 37 °C/5% CO_2_, and non-migrated or non-invaded cells were removed from the chamber interior by a cotton swab. Attached cells to the lower surface of the chamber were stained using Diff-Quick Kit (Sysmex, Kobe, Japan). Migrated or invaded cells were quantitated by dissolving stained cells in 100 μl of 10% acetic acid and then the mixture was transferred to a 96-well plate for colorimetric reading at 620 nm.

### Quantitative reverse transcription PCR (RT-qPCR)

The mRNA expression levels in breast cancer cells were detected by RT-PCR for IL-32θ and RT-qPCR for other target genes. Total RNA was isolated using Easy-BLUE (iNtRON Biotechnology, SungNam, Korea), then reverse transcription was performed. qPCR was conducted using SensiFAST™ SYBR NO-ROX Kit (BIOLINE, London, UK). Samples were analyzed using the primer sets listed in Additional file 1: Table S1. Transcript levels were quantitated using the -ΔCt method (Ct = fluorescence threshold value; −ΔCt = Ct GAPDH – Ct target gene).

### Enzyme-linked immunosorbent assay (ELISA)

Cells were cultured in the absence or presence of CM for 24 h, and then the culture media were replaced by fresh media for another 24 h. The cell culture supernatants were collected and analyzed using ELISA kits (R&D Systems, Minneapolis, MN) for human IL-1β, CCL5, CCL18, GM-CSF according to manufacturer’s instructions.

### Immunoblotting and immunoprecipitation

For nuclear and cytoplasmic fractionation, cells were collected and fractionated using the NE-PER kit (Thermo Fisher Scientific, Waltham, MA) according to manufacturer’s instructions. For immunoprecipitation, cell lysates were mixed with specific antibodies and then pulled down by protein G-agarose beads. Samples were subjected to 10% SDS–PAGE before being transferred to PVDF membranes (MilliporeSigma). The membranes were blocked with 5% skim milk dissolved in Tris-buffered saline containing 0.05% Tween-20 followed by primary antibody incubation at 4 °C overnight. After washing, horseradish peroxidase-conjugated IgG antibodies were added, and the membranes allowed to incubate for 1 h. Western blot was visualized using a chemiluminescence detection kit (Advanstar, Cleveland, Ohio) and detected by EZ-capture MG protein imaging system (ATTO, Tokyo, Japan). Specific antibodies used include those against Myc-tag, Flag-tag and phosphotyrosine-STAT3 (MilliporeSigma); IκBα, p-IκBα, p65, p50, PARP, and E-cadherin (Cell Signaling Technology, Danvers, MA); STAT3, COX-2, GAPDH (Santa Cruz Biotechnology, Dallas, TX); and anti-CCL18 neutralizing antibody (Abcam, Cambridge, MA). The monoclonal antibody KU-32-52 to detect IL-32 was prepared as previously described [20]. The raw data of western blot results can be seen in Additional file 2.

### Gelatin zymography

Cells (3 × 10^5^ cells/well) were seeded in a 6-well plate, cultured overnight, and then treated with or without CM for 24 h. MMP-9 activity in the supernatant was assayed as previously described [21]. Gel staining was conducted with InstantBlue™ (MilliporeSigma) for 30 min in the dark. Areas of gelatinolytic degradation appeared as transparent bands on the blue background.

### Immunofluorescence

Cells were seeded on coverslips and incubated overnight. The attached cells were fixed, and permeabilized with cold acetone before blocking with 0.1% bovine serum albumin in PBS at room temperature (RT). Primary antibodies were added (1:100) to the coverslip incubating at 4 °C overnight. After washing with PBS, the coverslips were incubated with secondary antibodies at (1:200). Nuclei staining was performed by exposing to 4, 6-diamidino-2-phenylindole (1:2000) (MilliporeSigma) for 20 s. The stained cells were visualized using an upright fluorescence microscope (Olympus, Tokyo, Japan).

### Xenograft model and optical imaging

All animal procedures were conducted according to the guidelines of the Institutional Animal Care and Use Committee (IACUC No. KU17008) of Konkuk University. MDA-MB-231-EV and MDA-MB-231-IL-32θ cells (5 × 10^6^ cells) were subcutaneously injected with Matrigel into the flanks of 5-week-old female athymic BALB/c nude mice (Nara Bio, Seoul, Korea). After 35 days, the tumors were harvested from euthanized mice. The tumor tissues were fixed with 10% formalin buffer, embedded in paraffin, and sectioned in 3 μm thickness for use in immunohistochemical analyses. Tumor volume was calculated using the formula V(mm^3^) = (shortest side^2^ × longest side)/2. For pre-and intra-operative tumor localization in the real-time resection, we conducted an in vivo tumor localization assay using the IRDye®-800CW 2-DG (2-deoxy-D-glucose) optical probe (LI-COR Biosciences, Lincoln, NE). Tumor localization was detected using optical imaging, particularly in the near-infrared fluorescence range. The tumorigenicity of MDA-MB-231-EV and MDA-MB-231-IL-32θ cells was assayed by intravenous injection of 1 × 10^6^ cells resuspended in PBS into nude mice (*n* = 5 per group).

### Immunohistochemistry

Formalin-fixed, paraffin embedded tumor tissue sections from mice were immersed in citrate buffer and boiled for 4 min in a microwave to retrieve antigens. Endogenous peroxidase activity was blocked with 3% (v/v) H_2_O_2_ for 10 min. Non-specific binding sites were blocked with 1% BSA for 30 min. Sections were incubated with the appropriate primary antibodies at 4 °C overnight and then the appropriate secondary antibodies for 1 h at RT. Diaminobenzidine tetrahydrochloride (Vector Laboratories, Burlingame, CA) was used as a substrate, and the sections were then counterstained with hematoxylin (MilliporeSigma).

### Statistical analysis

Chi-square or Fisher’s exact test was used to evaluate the relationship between IL-32θ expression and clinicopathological status. The mRNA expression in tumor tissues and protein secretion in breast cancer patients’ sera were analyzed by Mann-Whitney U test. Student’s *t*-test were used to compare the two groups in in vitro and in vivo experiments. Statistical analyses were performed using GraphPad Prism software version 5.0. All *p*-values were two-sided, and *p* < 0.05 was interpreted as being statistically significant.

## Results

### Association between tumor IL-32θ mRNA levels and breast tumor characteristics

To investigate whether IL-32θ was expressed in the tissues of breast cancer patients, we performed RT-PCR analyses using our specific primers as described [17]. Of the total of 90 breast tumors examined, 35 tumors expressed IL-32θ. The clinicopathological features and IL-32θ expression profiles for all patients are summarized in Table 1. IL-32θ expression was associated with tumor status, estrogen receptor (ER), progesterone receptor (PR), human epidermal growth factor receptor 2 (HER-2) status, and molecular classification characteristics. Interestingly, IL-32θ appeared frequently in ER negative, PR negative, HER-2 negative patients, and in those with triple negative-related breast cancer types (basal-like). Due to the lack of number of breast cancer patients with high metastasis status, the relationship between IL-32θ and this status could not be assessed accurately. In general, IL-32θ seemed to express in early tumor stage and be related triple negative breast cancer types.

**Table 1:** Association of IL-32θ mRNA expression and clinical characteristics of breast cancer patients

Characteristic	Total	IL-32θ expression		*P*-value
		Positive *n* (%)	Negative *n* (%)	
	*n* = 90	*n* = 35	*n* = 55	
Age				
> 60	9	4 (44.4)	5 (55.6)	0.7553^b^
≤ 60	81	31 (38.3)	50 (61.7)	
Tumor status				
T0–1	29	16 (55.1)	13 (44.8)	0.0361^a^
T2–3	61	19 (31.1)	42 (68.9)	
Nodal status				
N0–1	74	28 (37.8)	46 (62.2)	0.8036^a^
N2–3	16	7 (43.8)	9 (56.3)	
Metastasis status				
Yes	3	0 (0)	3 (100)	0.1674^b^
No	87	35 (40.2)	52 (59.8)	
Estrogen receptor (ER)				
Positive	64	18 (28.1)	46 (71.9)	0.001^a^
Negative	26	17 (65.4)	9 (34.6)	
Progesterone receptor (PR)				
Positive	53	14 (26.4)	39 (73.6)	0.0037^a^
Negative	37	21 (56.8)	16 (43.2)	
Human epidermal growth factor receptor 2 (HER-2)				
Positive	56	17 (30.4)	39 (69.6)	0.0331^a^
Negative	34	18 (52.9)	16 (47.1)	
Molecular classification				
Luminal A (ER+ PR+/− HER-2- Ki67 low)	20	9 (45)	11 (55)	0.04^a^
Luminal B (ER+ PR+/− HER-2+/Ki67 high)	44	11 (25)	33 (75)	
Basal-like (ER- PR- HER-2- EGFR+/Ki67 high)	14	9 (64.3)	5 (35.7)	
HER2-enriched (ER-PR-HER-2+ Ki67 high)	12	6 (50)	6 (50)	

Data are presented as number of patients. EGFR, epidermal growth factor receptor. ^a^Chi square test. ^b^Fisher exact test

### Opposing expression patterns of IL-32θ and CCL18 in breast tumor tissues

**Fig. 1:**
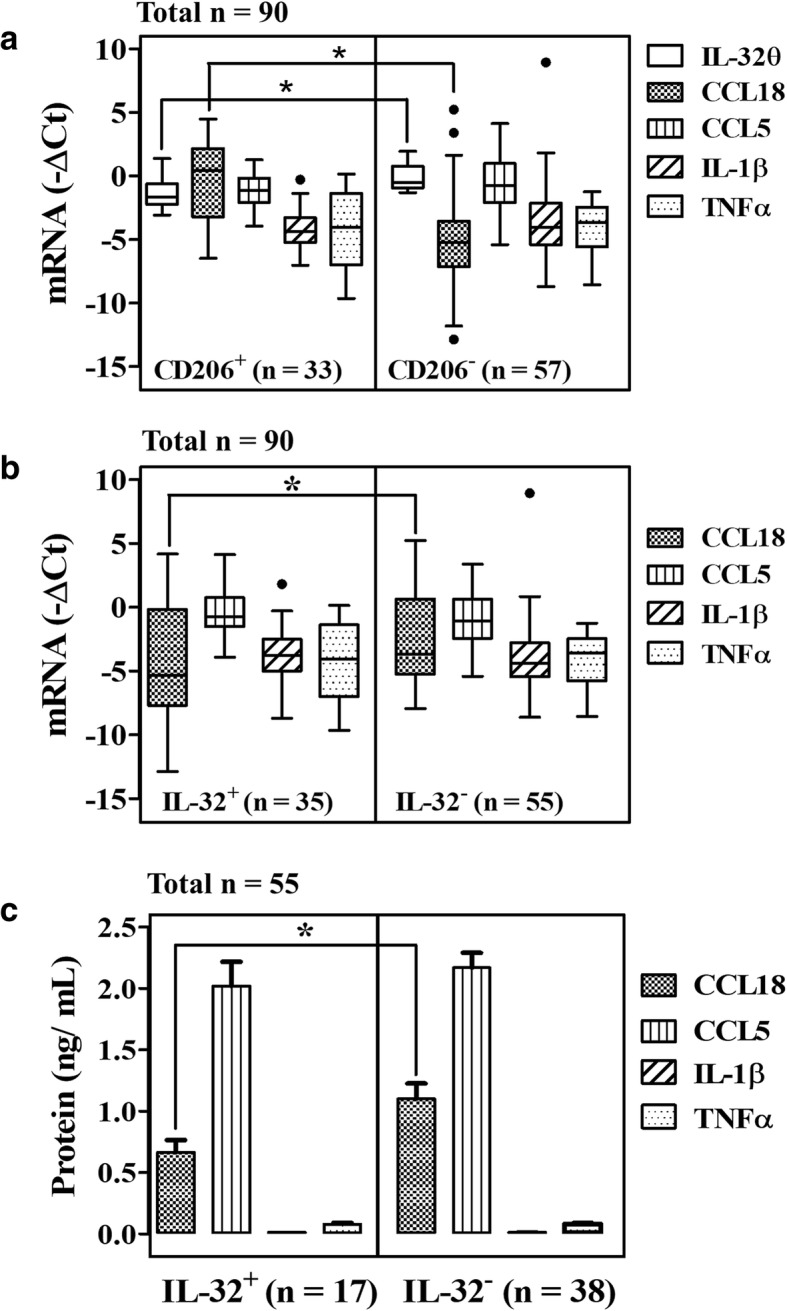
Opposing expression patterns between IL-32θ and CCL18 in selected tumor tissues. The mRNA expression levels of IL-32θ in tumor tissues were determined by RT-PCR, and then quantitated using ImageJ software. mRNA expression levels of CCL-18, IL-1β, TNF-α, and CCL5 were quantitated by real-time PCR. **a** mRNA expression of IL-32θ in CD206 positive (*n* = 33) and negative (*n* = 57) tumor tissue groups. **b** mRNA expression in IL-32 positive (*n* = 35) and negative (*n* = 55) tumor tissue groups. **c** Protein secretion level of CCL18, IL-1β, TNF-α, and CCL5 in IL-32 positive (*n* = 17) and negative (*n* = 38) tumor tissue groups. Plot are box and whisker plots. A line drawn across the box represents the median. Statistics were analyzed using Mann-Whitney U test: *, *p* < 0.05

Among the factors secreted by macrophages, CCL18 was reported to have strong effects on breast cancer progression whereas macrophage-secreted IL-1β, TNF-α, and CCL5 were previously suppressed by IL-32θ [12, 18, 22, 23]; thus, mRNA expression levels of these factors were measured. To identify the relationship between IL-32θ and breast cancer under the effect of TAMs, we divided the breast tumor tissues in two groups according to CD206 expression (an M2 macrophage marker), with a CD206^+^ status (*n* = 33) and CD206^−^ tissues (*n* = 57) and measured CCL18, IL-1β, TNF-α, and CCL5 mRNA by RT-qPCR (Fig. 1a). The results showed that CCL18 mRNA expression was significantly higher in in CD206^+^ group compared to CD206^−^ group in opposition to IL-32θ expression (*p* < 0.05), whereas IL-1β, TNF-α, and CCL5 showed no difference between two groups (Fig. 1a). To clarify this relationship, the IL-32θ^+^ patient group (*n* = 35) and IL-32θ^−^ patient group (*n* = 55) were further assessed (Fig. 1b). Additionally, of the 55 serum samples collected from breast cancer patients, protein secretion was measured in two groups IL-32θ^+^ patients (*n* = 17) and IL-32θ^−^ patients (*n* = 38) (Fig. 1c). Results indicated that in the presence of IL-32θ, CCL18 expression levels were lower than those without IL-32θ while IL-1β, TNF-α, and CCL5 levels showed no difference between two groups. Unfortunately, secreted IL-1β and TNF-α were detected at very low level in the sera (Fig. 1c). These findings suggest that higher IL-32θ expression in tumor tissue is accompanied by lower accumulation of CCL18 expression and vice versa while IL-1β or TNF-α or CCL5 expression are not affected by IL-32θ.

### IL-32θ reduces macrophage-regulated EMT, invasion, and migration in breast cancer cells in vitro

**Fig. 2:**
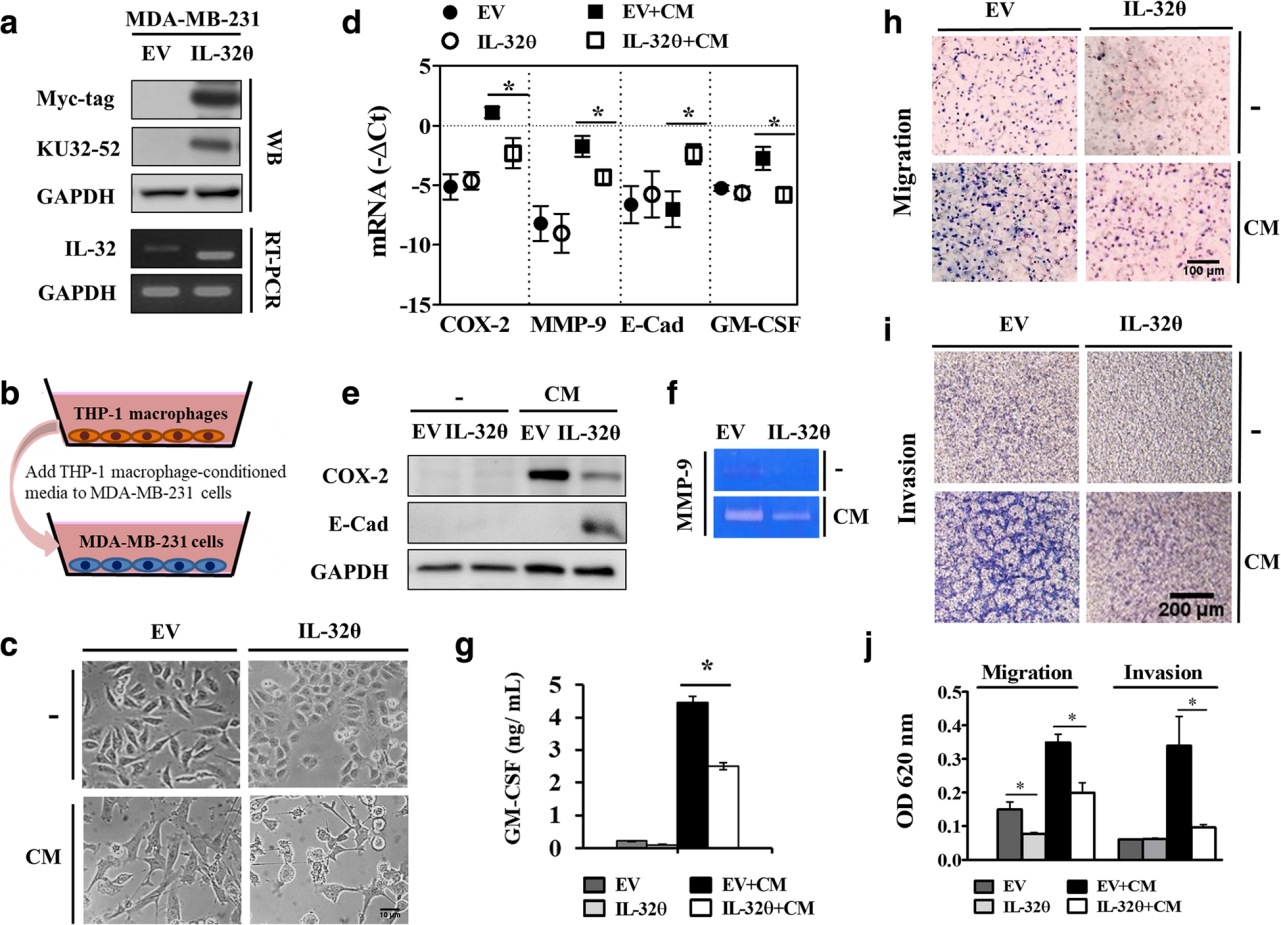
IL-32θ reduces breast cancer EMT, migration, invasion, and pro-malignancy factors in the CM treatment. **a** Constitutive expression system of 6x Myc-tagged IL-32θ in MDA-MB-231 cells by western blot and RT-PCR. **b** Schematic of in vitro model using MDA-MB-231-EV and MDA-MB-231-IL-32θ cells treated with CM from THP-1-derived macrophages. **c** Cellular morphological change in MDA-MB-231 EV and MDA-MB-231-IL-32θ cells in the absence (upper panel) or presence (lower panel) of CM. **d** mRNA expression levels of pro-malignancy factors in breast cancer cells were determined by real-time PCR (*n* = 5). **e** COX-2 and E-cadherin protein expression was analyzed by western blotting. **f** MMP-9 expression was detected by zymography. **g** Protein secretion levels of GM-CSF were measured by ELISA (*n* = 3). **h** and **i** The invasion or migration abilities of cells were performed using Matrigel-coated or non-coated transwell chambers. Related images were obtained from an upright microscope. **j** Migration or invasion intensities were quantitated based on an OD at 620 nm (*n* = 3). Scale bar, 10 μm (**c**); 100 μm (**h**); and 200 μm (**i**). All data are presented as the mean ± SEM and are analyzed using the Student’s *t*-test: *, *p* < 0.05. Western blot or RT-PCR or zymography images are the representative results of three independent experiments

MDA-MB-231, a triple negative breast cancer cell line, has mesenchymal-like phenotype and can undergo EMT to be more aggressive during tumor progression [24]; thus, we generated an MDA-MB-231 cell line stably expressing IL-32θ to study the function of IL-32θ in EMT. Due to the endogenous IL-32β expression in MDA-MB-231 cells [25], we used a specific primer set to recognize IL-32β and IL-32θ as described previously [17]. The IL-32θ PCR product appeared at 299 bp, whereas the other isoforms appeared at 360 bp because the IL-32θ sequence does not include exon 6, which is contained in IL-32β (Fig. 2a). PMA-treated THP-1 macrophages were reported to show equivalent properties to M2 macrophages [26], and this similarity was confirmed in the present study (Additional file 1: Figure S1a–e). Based on this phenomenon, CM from PMA-treated THP-1 macrophages was used to stimulate MDA-MB-231 progression (Fig. 2b). The cellular morphology of MDA-MB-231 stably expressing IL-32θ was observed without any stimulation, showing a more epithelial-like phenotype (Fig. 2c. upper panel). Consistent with previous research [12], MDA-MB-231 cells under CM treatment showed a more elongated shape and a mesenchymal-like phenotype compared to those in the non-treatment condition; however, IL-32θ still moderated the morphological change to a more epithelial-like state (Fig. 2c. lower panel). Hence, it is assumed that IL-32θ could potentially disrupt breast cancer EMT, invasion, and migration. For this reason, we evaluated whether IL-32θ could regulate the epithelial marker, E-cadherin, and other tumor-promoting factors, COX-2 and MMP-9, stimulated by macrophages. As a result, the expression of E-cadherin under the stimulation of CM was downregulated in MDA-MB-231 EV cells as expected when MDA-MB-231 EV cells underwent EMT to become more aggressive, whereas it was significantly upregulated in MDA-MB-231-IL-32θ cells (Fig. 2d-e). Significant downregulation of COX-2 and MMP-9 expression at the mRNA (Fig. 2d) and protein levels (Fig. 2e) were observed in MDA-MB-231-IL-32θ cells as compared to MDA-MB-231 EV cells with or without CM stimulation. A decreasing pattern was also observed regarding MMP-9 enzyme activity visualized by zymography (Fig. 2f). Moreover, macrophage-derived CCL18 was reported to create a feedback loop between macrophage and breast cancer cells by stimulating breast cancer-derived GM-CSF [12]. In this study, the GM-CSF mRNA and secretion levels were found to be significantly upregulated in the presence of CM, which was markedly inhibited by IL-32θ (Fig. 2d, g). To further determine the effects of IL-32θ on cancer progression features, a transwell migration assay and a Matrigel invasion assay were performed (Fig. 2h-i). In the presence of CM, the stimulated MDA-MB-231-EV cells displayed increased rates of migration and invasion, and these rates were significantly reduced in MDA-MB-231-IL-32θ cells (Fig. 2j). These data supported the role of IL-32θ in suppressing macrophage-induced breast cancer progression.

### IL-32θ directly interacts with PKCδ to subsequently inhibit NF-κB and STAT3 pathways in vitro

**Fig. 3:**
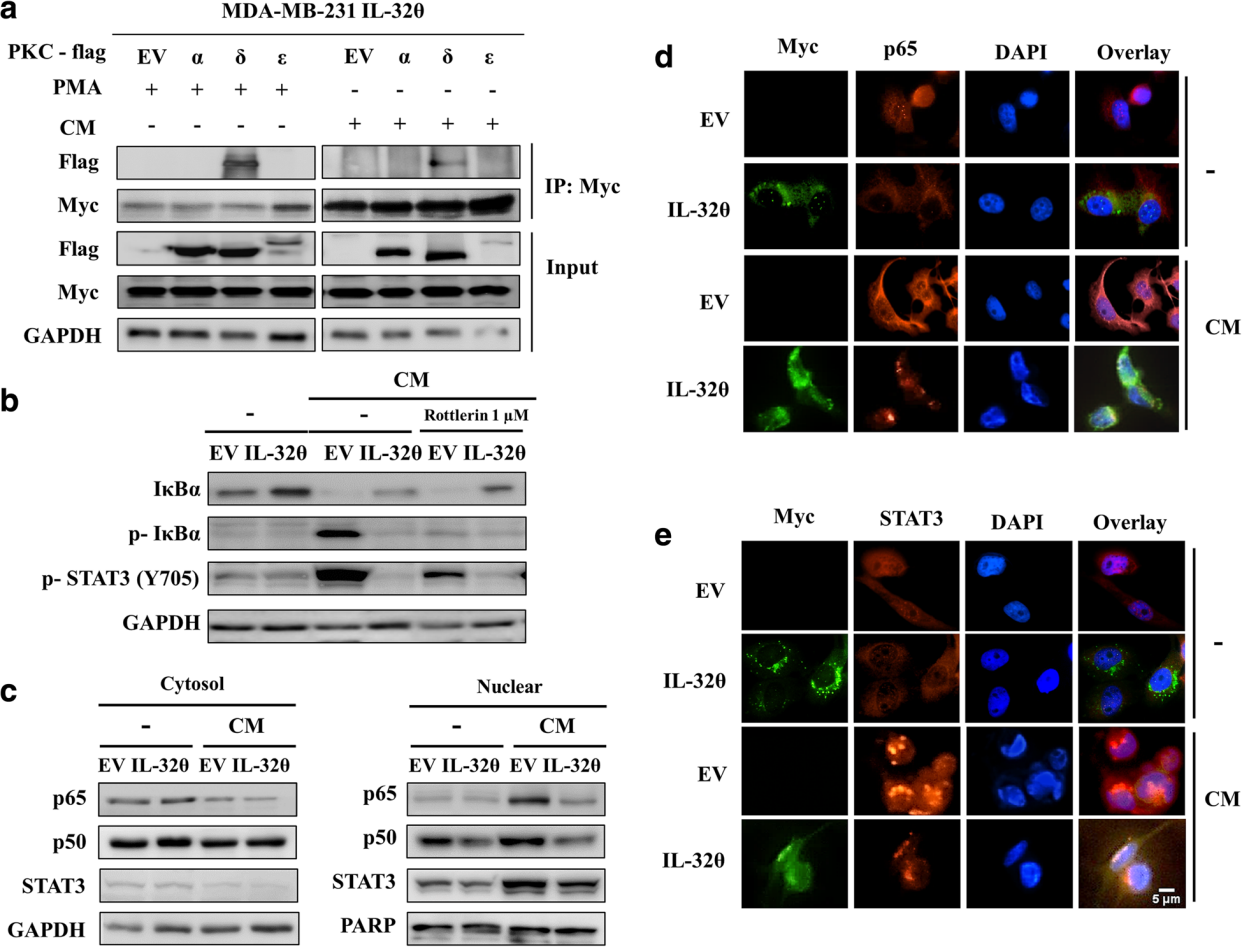
IL-32θ interacts with PKCδ, and subsequently inhibits the NF-κB and STAT3 pathways in MDA-MB-231 cells. **a** IL-32θ interacts with PKCδ upon stimulation with PMA or CM. Immunoprecipitation was performed using anti-Myc antibody. **b-c** MDA-MB-231-EV and MDA-MB-231-IL-32θ cells in the absence or presence of CM of THP-1 macrophages were harvested and separated into cytosol and nuclear fractions. NF-κB nuclear translocation and phosphorylation of IκBα and STAT3 was detected by western blot. **d-e** Immunofluorescence assay to detect p65 (**d**) or STAT3 (**e**) (red) and Myc-IL-32θ (green) localization (scale bar, 5 μm). Nuclei were stained with DAPI (blue). Western blot or immunofluorescence images are the representative results of three independent experiments

To investigate the precise mechanism by which IL-32θ might regulate breast cancer progression, the relationship between IL-32θ and PKCδ in breast cancer cells was explored based on previous studies demonstrating their interaction [16, 19]. An immunoprecipitation assay showed that IL-32θ could interact with only PKCδ upon PMA activation or CM stimulation in MDA-MB-231 IL-32θ cells (Fig. 3a). Therefore, it was hypothesized that IL-32θ interacted with PKCδ upon stimulation with THP-1 macrophage CM to subsequently modulate downstream pathways in breast cancer cells. Based on the transcription factors inhibited by IL-32θ previously [17–19], NF-κB and STAT3 were assumed to be involved in the IL-32θ-mediated PKCδ signaling. The western blot results revealed that in both CM treatment and non-treatment condition, IL-32θ elevated IκBα expression, and inhibited phosphorylation of IκBα and STAT3 at tyrosine 705 (Fig. 3b). Next, the nuclear translocation levels of STAT3, p65 plus p50 (two subunits of NF-κB), which were increased remarkably in the treatment with CM, were downregulated by IL-32θ (Fig. 3c). Further, immunofluorescence analysis was performed to determine the location of IL-32θ, p65, and STAT3 in the nucleus and cytoplasm of MDA-MB-231 cells. The results were consistent with the western blot data, revealing that the fluorescence accumulation of p65 and STAT3 in the nucleus after stimulation with the CM was strongly reduced in IL-32θ-expressing cells (Fig. 3d-e). To confirm that IL-32θ regulated NF-κB and STAT3 through PKCδ, a pharmacological inhibition of PKCδ signaling by rottlerin [27] was applied before macrophage CM treatment. It was indicated that the PKCδ inhibitor could suppress IκBα degradation and STAT3 phosphorylation, and additional effects of rottlerin and IL-32θ on these signals were also observed (Fig. 3b). These data demonstrated that PKCδ mediated NF-κB and STAT3 signaling, and IL-32θ inhibited these pathways.

### Blocking CCL18 signaling downregulates pro-malignancy factors and the PKCδ downstream pathway

**Fig. 4:**
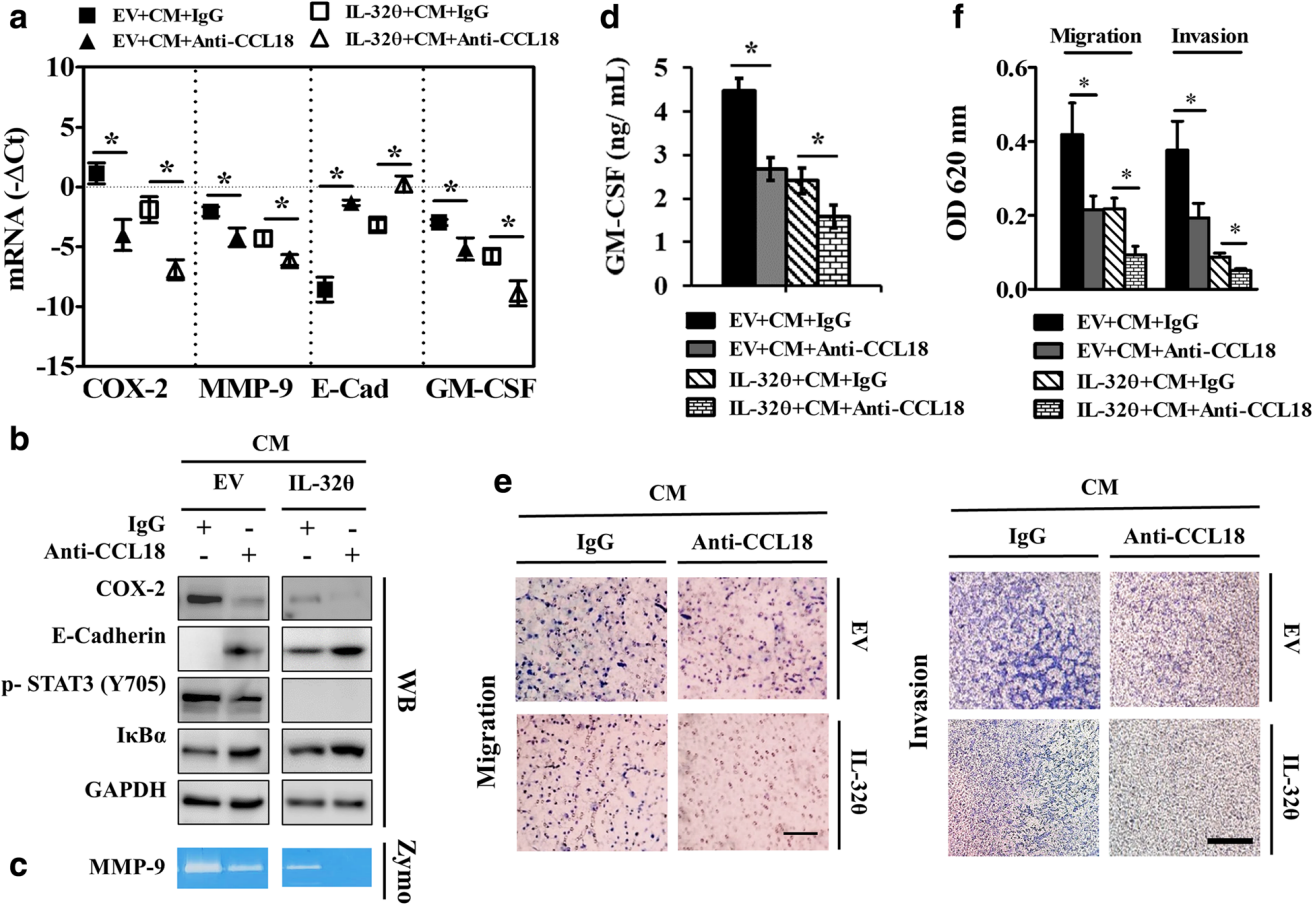
Blocking CCL18 signaling reduces PKCδ-mediated signaling and metastasis-related factors. MDA-MB-231-EV cells were treated with CM in the absence or presence of CCL18 neutralizing antibodies. IgG antibody was used as a negative control. **a** mRNA expression levels of pro-malignancy factors were determined by real-time PCR (*n* = 5). **b** COX-2, E-cadherin, phosphor-STAT3 (Y705), and IκBα protein expression was analyzed by western blotting. **c** MMP-9 expression was detected by zymography. **d** Protein secretion of GM-CSF was measured by ELISA (*n* = 3). Western blot or zymography images are the representative results of three independent experiments. **e** The invasion or migration abilities of cells were performed using Matrigel-coated or non-coated transwell chambers. Related images were obtained from an upright microscope. **f** Migration and invasion intensities were quantitated based on an OD at 620 nm (*n* = 3). Scale bar, 100 μm (migration) and 200 μm (invasion). All data are presented as the mean ± SEM and are analyzed using the Student’s *t*-test: *, *p* < 0.05

The secretion levels of CCL18 were significantly detected in the supernatant of THP-1-derived macrophage activated by PMA compared to the untreated control (Additional file 1: Figure S1f). Therefore, it was assumed that macrophage-secreted CCL18 might play regulating roles in EMT, invasion, and migration through PKCδ signaling which was suppressed by IL-32θ. To support this idea, CCL18 signaling was blocked using a neutralizing antibody in co-treatment with CM in both MDA-MB-231 EV cells and MDA-MB-231 IL-32θ cells. The disappearance of CCL18 signaling in MDA-MB-231 EV cells significantly downregulated the expression of COX-2, MMP-9, GM-CSF, and upregulated E-cadherin at both mRNA and protein levels (Fig. 4a–d). Moreover, the degradation of IκBα, which represented the PKCδ downstream signaling, NF-κB, was strongly reduced while the phosphorylated STAT3 at tyrosine 705 was slightly downregulated in the absence of CCL18 in MDA-MB-231 EV cells (Fig. 4b). Furthermore, the absence of CCL18 in CM impaired the effects of CM on the migration and invasion rates of MDA-MB-231 EV cells (Fig. 4e-f). These data suggest that CCL18 acts as an upstream activator of PKCδ signaling (including two downstream pathways, NF-κB and STAT3) to induce breast cancer progression. The cytokine CCL18 seemed to influence NF-κB, and partly through STAT3 to stimulate E-cadherin, COX-2, MMP-9, and GM-CSF expression. Notably, IL-32θ might collaborate with neutralizing CCL18 antibody to display additive effects in the decrease of the expression of metastasis-related factors in the MDA-MB-231 IL-32θ cells as compared to that in the MDA-MB-231 EV cells (Fig. 4a-d). The results from migration and invasion assays, which were performed after blocking CCL18 signaling, indicated that the migration and invasion rates of the MDA-MB-231-IL-32θ cells were reduced to the minimum when compared to the IgG treated control group (Fig. 4 e-f). Therefore, it can be confirmed that CCL18 signaling is the main target of IL-32θ to inhibit the macrophage-induced metastasis of breast cancer cells.

### IL-32θ inhibits tumor formation of breast cancer cells in vivo

**Fig. 5:**
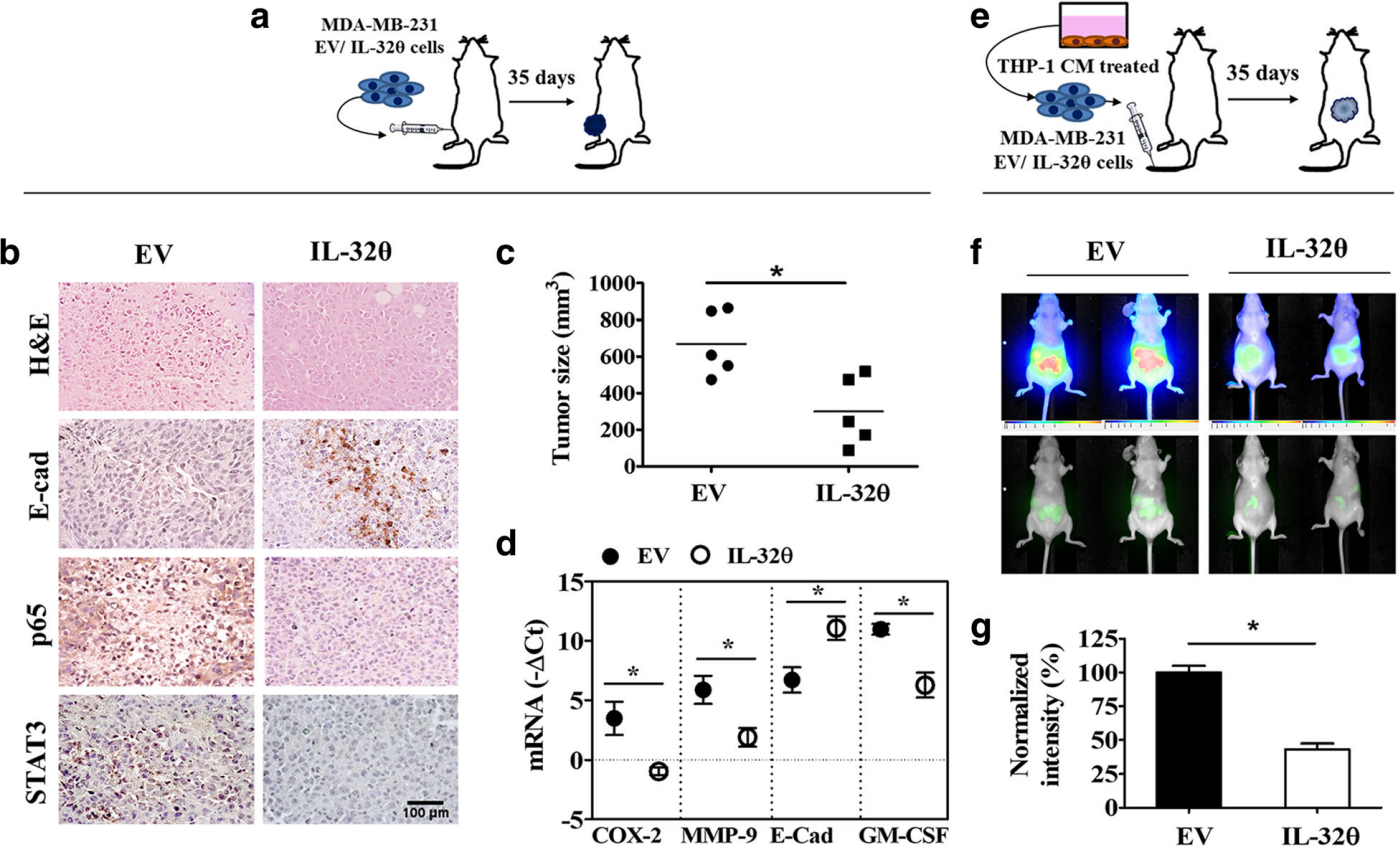
IL-32θ inhibits tumor formation in a breast cancer xenograft model. **a** Schematic of mouse model 1: MDA-MB-231-EV and/or MDA-MB-231-IL-32θ cells were injected into the flanks of mice (*n* = 10/ group). Tumors from two mouse groups were extracted after 35 days and analyzed. **b** Images are presented from hematoxylin-eosin (HE) staining and immunohistochemistry staining of section from tumors. Immunohistochemistry images are the representative of ten sections. **c** Tumor size was measured after 35 days. **d** mRNA expression levels of pro-malignancy factors were quantitated by real-time PCR. **e** Schematic of mouse model 2: MDA-MB-231-EV and/or MDA-MB-231-IL-32θ cells were treated with THP-1 CM for 24 h then probe administrated for 24 h before intravenous injection into mice (*n* = 5/ group). **f** After 35 days, tumor localization was analyzed by IRDye®-2DG infrared optical probe-guided analysis. Representative imaging data of breast cancer cell-induced solid tumors in mice. **g** Fluorescence intensities were obtained by ImageJ software. Scale bar, 100 μm. Data are presented as the mean ± SEM and are analyzed using the Student’s *t*-test: *, *p* < 0.05

To examine the cancerous properties of MDA-MB-231-EV or MDA-MB-231-IL-32θ cells, cells were injected to the flanks of mice in xenograft model (Fig. 5a). The immunohistochemistry results showed that the intensity of E-cadherin-positive cells was significantly increased, whereas the intensity for p65 and STAT3-positive cells were relatively decreased in the tumor tissue of the MDA-MB-231-IL-32θ group compared to that of the MDA-MB-231-EV group (Fig. 5b). Moreover, tumor volume was reduced significantly in the mouse group injected with MDA-MB-231-IL-32θ cells (Fig. 5c). Furthermore, the mRNA levels of various tumor-promoting factors including COX-2, MMP-9, E-cadherin, and GM-CSF were down-regulated in the MDA-MB-231-IL-32θ group (Fig. 5d). These findings supported the idea that the antitumor activity of IL-32θ was associated with the inactivation of NF-κB and STAT3 in tumor tissues. In another model, the MDA-MB-231-EV or MDA-MB-231-IL-32θ cells were treated with macrophage CM for 24 h before intravenous injection to nude mice (Fig. 5e). As shown in Fig. 5f-g, MDA-MB-231-EV cell tumors were large and emitted strong fluorescence signal, whereas a weaker pattern was observed in the mice treated with MDA-MB-231-IL-32θ cells, suggesting potential effects of IL-32θ on the tumor progression of stimulated breast cancer cells.

## Discussion

Macrophages, a major component of the tumor microenvironment, can initiate and support the tumor progression and metastasis by secreting a range of growth factors, cytokines, and chemokines [28]. IL-32 was found to not only target cancer cells but might also target the tumor microenvironment [29]. Recent reports showed the correlation and different functions of IL-32 and its isoforms to various cancer diseases. As an example, IL-32γ can inhibit colon cancer cell growth by targeting NF-κB and STAT3 pathways [30] while another isoform, IL-32β, stimulates the migration of breast cancer cells through VEGF-STAT3 [25], and is involved in the increase of glycolysis under hypoxic conditions which supports cancer cell growth [31]. Given these data, it appears that the effects of IL-32 on tumor development depend on both its isoforms and cancer types; however, the exact mechanisms remain unclear. Our previous data on IL-32θ, a recently discovered isoform, demonstrated its inhibition ability in macrophage differentiation [32], macrophage-secreted factors [16, 18, 19], and in colon cancer progression by regulating self-renewal and EMT [17]. In this study, three isoforms, IL-32θ, IL-32β, and IL-32γ, were detected at different mRNA levels in 90 breast tumors. IL-32β exhibited the strongest expression which was compatible with its protumor effects reported in breast cancer [25] while IL-32γ was rarely expressed compared to the IL-32θ isoform (Additional file 1: Fig. S2a-c). Given this, the current study attempted to discover the role of IL-32θ in breast cancer progression and its tumor microenvironment. Our clinical data showed that IL-32θ expression was associated with the negativity of ER, PR, and HER-2, and with triple negative related breast cancer types. Based on this point, we chose MDA-MB-231 cells, a highly aggressive, basal-like breast cancer cells with triple negative background [33], together with PMA-treated THP-1 macrophage cells to mimic the interaction between macrophages and cancer cells within the tumor microenvironment and evaluate the role of IL-32θ on this interaction in vitro. This basal-like cell line is associated with both a poor prognosis and clinical outcome, due to its aggressiveness and high rate of metastasis [34]. We determined that the EMT phenotypic changes of MDA-MB-231 cells caused by stimulation of CM from THP-1 macrophages could be inhibited by IL-32θ. Moreover, invasion and migration rates were remarkably reduced in IL-32θ-expressing cells after 24 h treatment with CM, suggesting that IL-32θ could be a potential factor inhibiting macrophage-induced breast cancer progression. The interaction between macrophages and breast cancer cells has been reported to increase levels of various tumor promoting factors such as COX-2, and MMP-9 which, in turn, supports the breast malignancy and an increase of TAM density in the tumor microenvironment [23, 26, 28, 35]. In agreement with these reports, the present study indicated that IL-32θ downregulated COX-2, MMP-9, and E-cadherin expression in breast cancer cells stimulated by macrophages demonstrating a modulatory role of IL-32θ in breast cancer development.

In addition, the precise mechanism by which IL-32θ reduces the effects of macrophage on breast cancer progression was addressed based on previous studies detailing that IL-32θ interacted directly with PKCδ to subsequently decrease STAT3 or NF-κB signaling in PMA-activated THP-1 cells [16, 19]. In line with this theory, the present study showed a direct interaction between IL-32θ and PKCδ in breast cancer cells. Especially under CM treatment condition, IL-32θ inhibited phosphorylation of IκBα plus STAT3, and nuclear translocation of NF-κB and STAT3 in MDA-MB-231 cells (Fig. 3b). Moreover, interfering PKCδ signaling with rottlerin, a PKCδ inhibitor, resulted in additive effects with IL-32θ in the decrease of STAT3 phosphorylation and IκBα degradation. Due to the fact that PKCδ mRNA expression was found to be significantly higher in ER-positive compared with ER-negative tumors [36], we applied this model on another breast cancer cell line with an estrogen-dependent background, MCF-7. However, IL-32θ could not reduce any signal activated by macrophage CM in MCF-7 cells (Additional file 1: Figure S3a-b). Since MCF-7 represents epithelial-like cells and MDA-MB-231 represents mesenchymal-like cells, it is suggested that IL-32θ seemed to effectively modulate the breast cancer with EMT-associated macrophages, which is essential for metastasis. The lack of PKCδ activation in MDA-MB-231 in the non-stimulated condition disappeared when MDA-MB-231 was co-cultured with macrophage CM. Further studies are necessary to define the association between IL-32θ and mesenchymal-like cells but not epithelial-like cells. In any case, these findings demonstrated that IL-32θ targeted the interaction between macrophage and mesenchymal-like breast cancer, and there requires a specific macrophage-secreted factor to trigger PKCδ signaling in breast cancer which was inhibited by IL-32θ.

During the investigation of the IL-32θ-regulated signaling upstream factors, CCL18 was considered as a potential activator due to its presence in the THP-1 macrophage CM, and the inverse expression between IL-32θ and CCL18 in breast tumor tissues infiltrated with CD206+ macrophages. GM-CSF secreted from breast cancer cells activates macrophages to become CCL18-expressing TAM-like cells, which reciprocally supports GM-CSF secretion and furthers EMT of breast cancer cells [12]. Moreover, only GM-CSF significantly induced the production of TAM-related cytokines, and GM-CSF was found in CM from MDA-MB-231 cells but not MCF-7 cells [12]. Consistent with this study, our study found a decrease in the amount of GM-CSF secretion in IL-32θ-expressing MDA-MB-231 cells which might be stimulated by CCL18 from macrophages. Further, IL-32θ did not suppress endogenous GM-CSF in MDA-MB-231 cells due to a lack of PKCδ activation and interaction. The transcription factors STAT3 and NF-κB were also reported as the downstream factors regulated by PKCδ in cancer cells [37, 38]. In line with the idea that CCL18 is a stimulator of PKCδ signaling, our data demonstrated that blocking CCL18 signaling suppressed the expression of the PKCδ downstream factors STAT3 or NF-κB as well as various cancer-related factors. These results supported the idea that macrophage-secreted CCL18 might act as a stimulator of PKCδ signaling regulated by IL-32θ.

The present study also provides the first in vivo evidence of the suppressive function of IL-32θ in breast cancer. A xenograft mouse model of MDA-MB-231-IL-32θ cells showed an increase of E-cadherin-positive cells, suggesting that IL-32θ reversed the effects on EMT, whereas STAT3 and NF-κB-positive cells were much more abundant in the absence of IL-32θ. Another imaging model in which breast cancer cells were activated by macrophage CM to become more aggressive also supported that IL-32θ could reduce the tumor localization clearly compared to the MDA-MB-231-EV group. These in vivo results are in accordance with the in vitro and clinical data demonstrating that IL-32θ acts via PKCδ signaling to regulate the effects of macrophage-soluble factors on breast cancer cells.

The small population of patients’ data collected recently does not allow us to perform a survival analysis to assess the relationship between IL-32θ and the survival rate of breast cancer patients. Moreover, during studying about the effects of IL-32θ on breast cancer cell proliferation, we have found that Bcl-2, an anti-apoptotic factor which has been proposed as a prognostic marker [39], was totally repressed by IL-32θ in vitro (data not shown). However, no significant change between two cell lines could be seen in the expression levels of the late apoptotic markers after 72 h from flow cytometry results (data not shown). Thus, it is necessary to study different types of cell death to understand by which mechanism IL-32θ may affect the cell death. Finally, although there are some aspects described above to be considered, these will be the subjects of ongoing studies.

## Conclusions

**Fig. 6:**
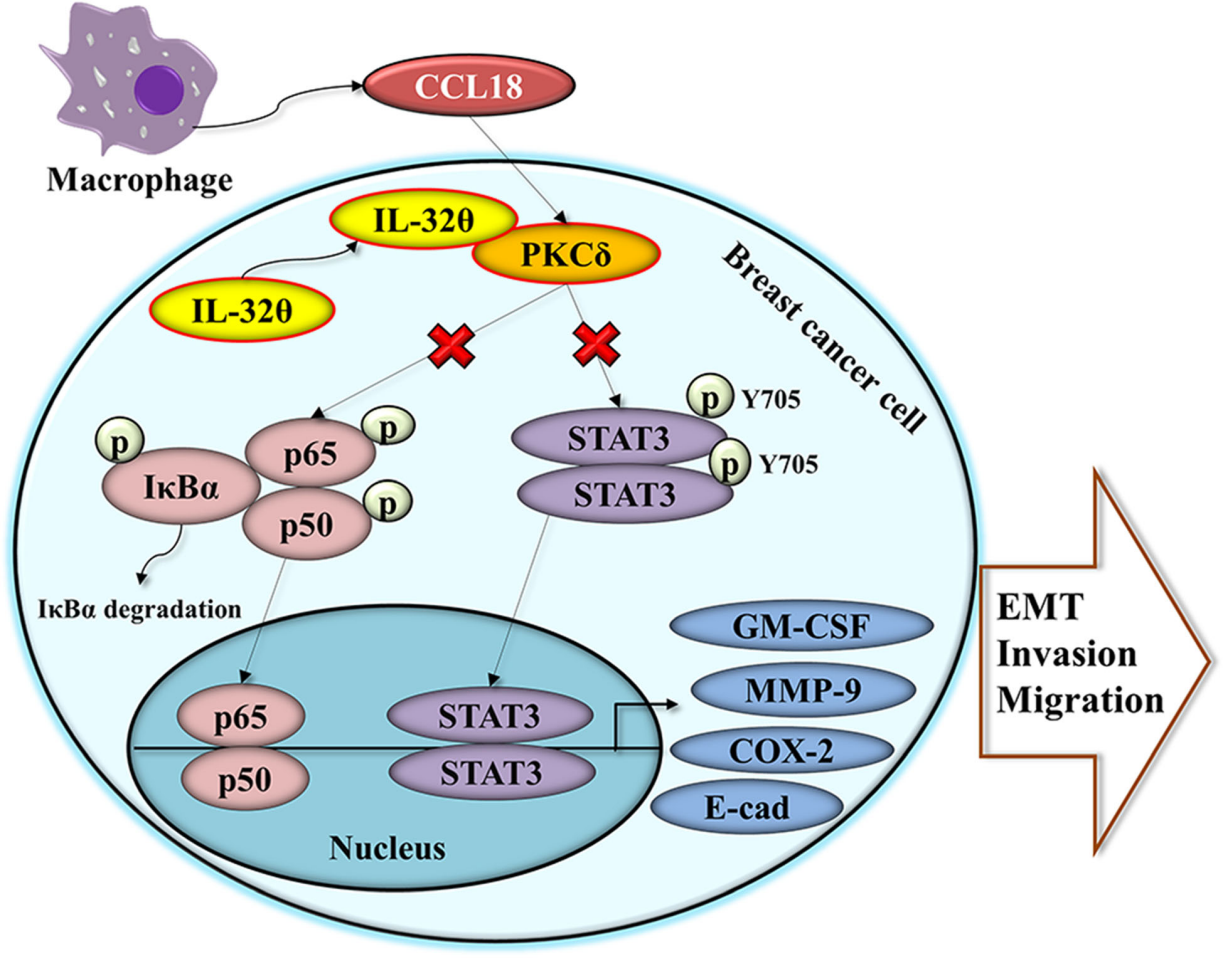
Schematic diagram of the signaling cascade inhibited by IL-32θ in breast cancer cells. In brief, THP-1-derived macrophages secrete pro-inflammatory factors such as CCL18 to stimulate PKCδ signaling, which can elevate the levels of epithelial-mesenchymal transition (EMT) invasion and migration in breast cancer cells. IL-32θ suppresses these factors by directly interacting with PKCδ to subsequently reduce NF-κB and STAT3 levels

In conclusion, IL-32θ inhibited EMT and metastasis in breast cancer cells by targeting CCL18 secreted from macrophages. A schematic diagram of this mechanism was shown in Fig. 6. The IL-32θ-mediated inhibition of macrophage-breast cancer cross-talk shows potential for a therapeutic strategy in blocking pro-metastatic activity of breast cancer.

## Additional files

Additional file 1: **Table S1.** PCR primer sequences. **Figure S1.** PMA-induced macrophages show an M2-like phenotype. **Figure S2.** mRNA expression of IL-32θ, IL-32β, IL-32γ in breast tumors. **Figure S3**. Effect of IL-32θ on pro-malignancy and signaling factors in macrophage CM-treated MCF-7-EV and MCF-7-IL-32θ cells. (PDF 514 kb)

Additional file 2: Raw data of Western blot. (PPTX 576 kb)

## Data Availability

All results of this study are presented in this article and additional files.

## Abbreviations

CCL: Chemokine (C-C motif) ligand
CD206: Cluster of differentiation 206
CM: Conditioned media
COX-2: Cyclooxygenase 2
EMT: Epithelial-mesenchymal transition
GM-CSF: Granulocyte-macrophage colony-stimulating factor
IL: Interleukin
MMP-9: Matrix metallopeptidase 9
PKC: Protein kinase C
STAT3: Signal transducer and activator of transcription 3
TAMs: Tumor-associated macrophages
