# Supplementary material for: Interleukin-32θ inhibits tumor-promoting effects of macrophage-secreted CCL18 in breast cancer
Source: Cell Commun Signal. 2019 May 24;17:53. doi: 10.1186/s12964-019-0374-y (PMC6534939; doi:10.1186/s12964-019-0374-y)
Supplement: Supplementary file 1 — Table S1. PCR primer sequences. Figure S1. PMA-induced macrophages show an M2-like phenotype. Figure S2. mRNA expression of IL-32θ, IL-32β, IL-32γ in breast tumors. Figure S3. Effect of IL-32θ on pro-malignancy and signaling factors in macrophage CM-treated MCF-7-EV and MCF-7-IL-32θ cells. (PDF 514 kb) [file 12964_2019_374_MOESM1_ESM.pdf]

Additional file 1

Table S1. PCR primer sequences.

**Genes**		**Primer sequences (5'→ 3')**	**References**
**GAPDH**	Forward	TGATGACATCAAGAAGGTGGT	Bak Y, *et al.* (2014)
	Reverse	TCCTTGGAGGCCATGTAGGCC	
**IL-32θ**	Forward	TGATGTCGAGCCTGGCAGAG	Kim MS, *et al.* (2015)
	Reverse	GAGCTCTGACAGAGAGCAGC	
**CD68**	Forward	GCTGGCTGTGCTTTTCTCG	Reuwer AQ, *et al.* (2011)
	Reverse	GTCACCGTGAAGGATGGCA	
**E-Cadherin**	Forward	GAAGGTGACAGAGCCTCTGGAT	Bak Y, *et al.* (2016)
	Reverse	ATCGGTTACCGTGATCAAAATC	
**MMP-9**	Forward	CGCAGACATCGTCATCCAGT	Song YS, *et al.* (2015)
	Reverse	GGATTGGCCTTGGAAGATGA	
**COX-2**	Forward	CAAATCCTTGCTGTTCCCACCCAT	Wu XL, *et al.* (2013)
	Reverse	GTGCACTGTGTTTGGAGTGGGTTT	
**IL-1β**	Forward	TACATCCTCGACGGCATCTCA	Kim MS, *et al.* (2014)
	Reverse	CTACATTTGCCGAAGAGCCCT	
**CCL5**	Forward	GCTGTCATCCTCATTGCTAC	Bak Y, *et al.* (2014)
	Reverse	CATTTCTTCTCTGGGTTGGC	
**GM-CSF**	Forward	AAATGTTTGACCTCCAGGAG	
	Reverse	TGATAATCTGGGTTGCACAG	
**CCL18**	Forward	TCTATACCTCCTGGCAGATTC	Gunther C, *et al.* (2011)
	Reverse	TTTCTGGACCCACTTCTTATTG	

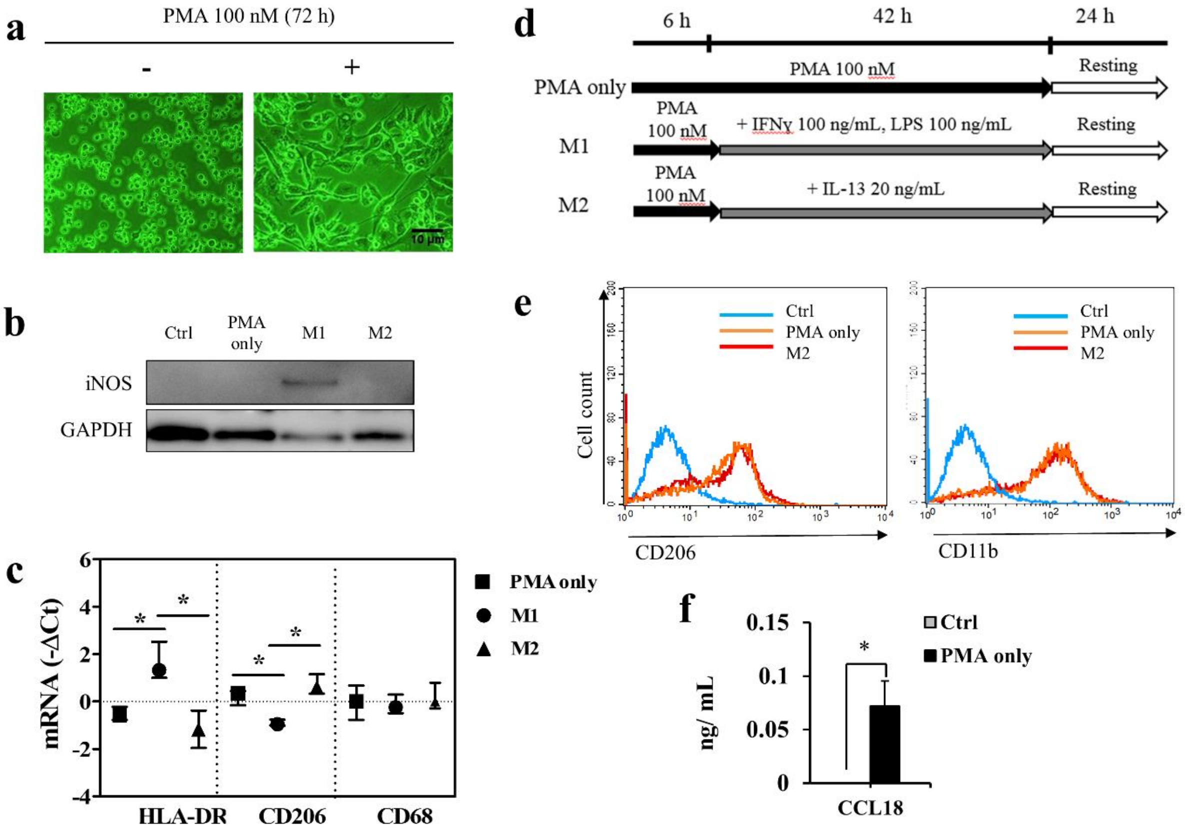

Figure S1. PMA-induced macrophages show an M2-like phenotype. a, Cellular morphological change of THP-1 cells after treatment with 100 nM PMA for 72 h. b, After treatment with 100 nM PMA for 6 h, THP-1 cells were either kept in PMA treatment conditions for an additional 42 h to differentiate into the "PMA-treated THP-1 macrophages" (labeled "PMA only"), or cultured with PMA plus 100 ng/ml LPS and 100 ng/ml IFN-γ for 42 h to become M1-polarized THP-1 macrophages (labeled "M1"), or cultured with PMA plus 20 ng/ml IL-13 for another 42 h to become M2-polarized THP-1 macrophages (labeled “M2”). Differentiated THP-1 cells were then cultured in fresh media for a further 24 h before being harvested for other experiments. c, The M1 macrophage marker iNOS was detected by western blot. d, The macrophage marker, CD11b, and M2 macrophage marker, CD206, were quantified via FACS analysis. e, Macrophage markers were quantitated by RT-qPCR. f, Secretion levels of CCL18 in THP-1 macrophages activated by PMA 100 nM were measured by ELISA and compared with those of the THP-1 control without any treatment. Results were from three independent experiments. Data are presented as the mean ± SEM and are analyzed using the Student’s *t*-test: *, $p < 0.05$.

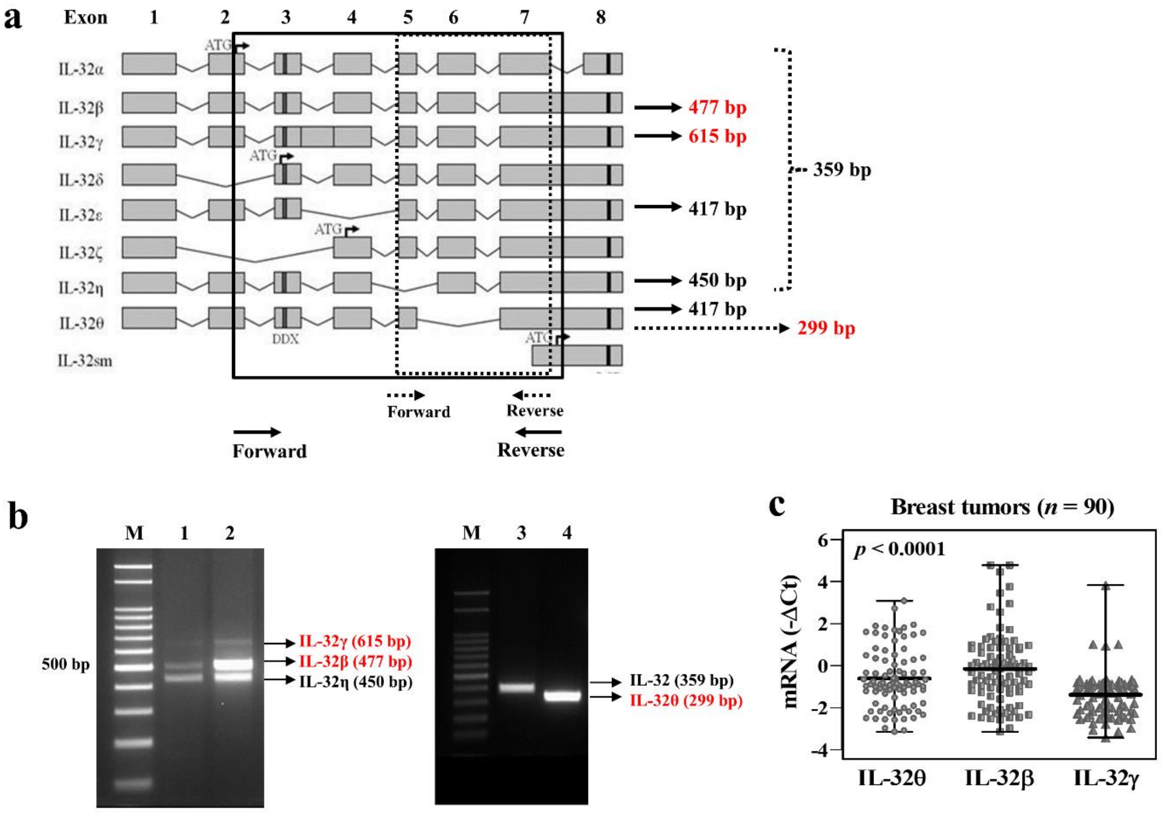

Figure S2. mRNA expression of IL-32θ, IL-32β, IL-32γ in breast tumors. **a** The primers (Forward primer: 5'-GGTCCTCTCTGATGACATGA-3'; Reverse primer: 5'-TGGAAAGAGGACATGAAGAG-3') targeting the sequence from exon 2 to exon 7 were designed to detect IL-32β (477 bp) and IL-32γ (615 bp). Primers to detect IL-32θ (299 bp) were used as previously described (Kim MS, *et al.*, 2015). **b** Example of mRNA expression from different IL-32 isoforms in breast tumors. **c** mRNA expression of IL-32θ, IL-32β, and IL-32γ in 90 breast tumors were performed by RT-PCR and quantitated by ImageJ software. Data are presented as the median with range and are analyzed using the Kruskal–Wallis test followed by Dunn's post hoc tests: $p < 0.0001$.

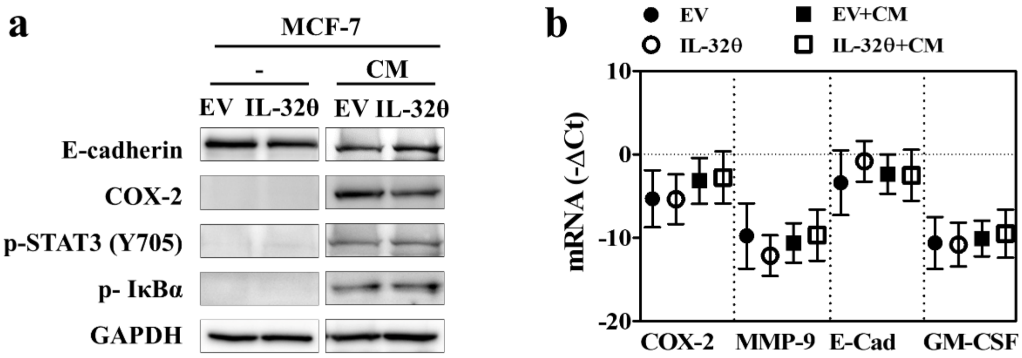

Figure S3. Effect of IL-32θ on pro-malignancy and signaling factors in macrophage CM-treated MCF-7-EV and MCF-7-IL-32θ cells. MCF-7 cells were transfected pcDNA3.1–6X Myc-IL-32θ (MCF-7-IL-32θ) or pcDNA3.1(+)-6X Myc (MCF-7-EV) for 48 h then cells were treated with macrophage CM for an additional 24 h before being harvested. **a** Protein expression of COX-2, E-cadherin, phosphorylated STAT3 (Y705), and phosphorylated IκBα were measured by western blotting. **b** mRNA expression was measured by RT-qPCR. Data are presented as the mean ± SEM and are analyzed using the Student's *t*-test: *, $p < 0.05$.
