# Supplementary material for: Interleukin-32θ inhibits tumor-promoting effects of macrophage-secreted CCL18 in breast cancer
Source: Cell Commun Signal. 2019 May 24;17:53. doi: 10.1186/s12964-019-0374-y (PMC6534939; doi:10.1186/s12964-019-0374-y)
Supplement: Supplementary file 2 — Raw data of Western blot. (PPTX 576 kb) [file 12964_2019_374_MOESM2_ESM.pptx]

## Slide 1
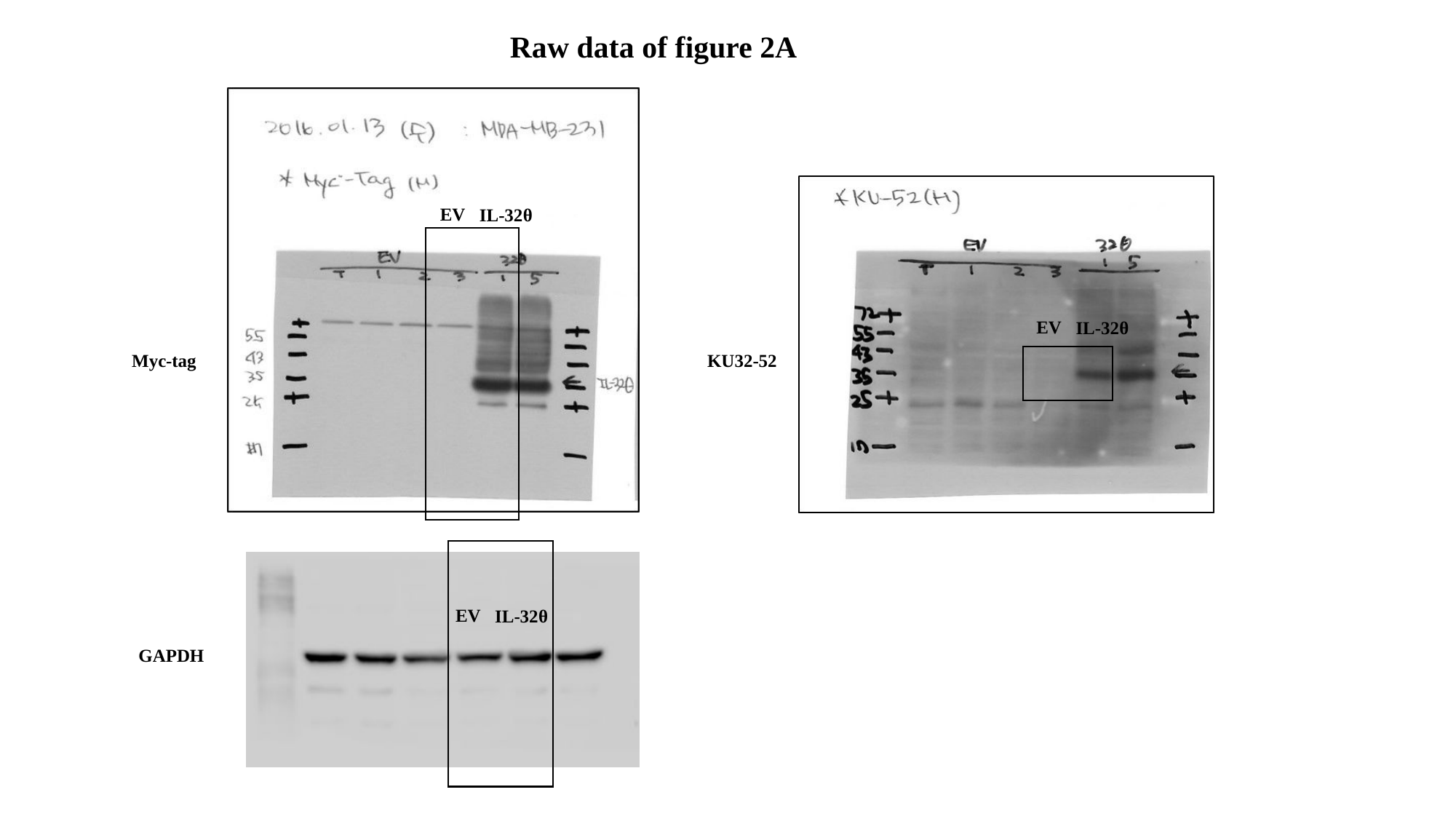

Raw data of figure 2A
EV
IL-32θ
EV
IL-32θ
Myc-tag
KU32-52
EV
IL-32θ
GAPDH

## Slide 2
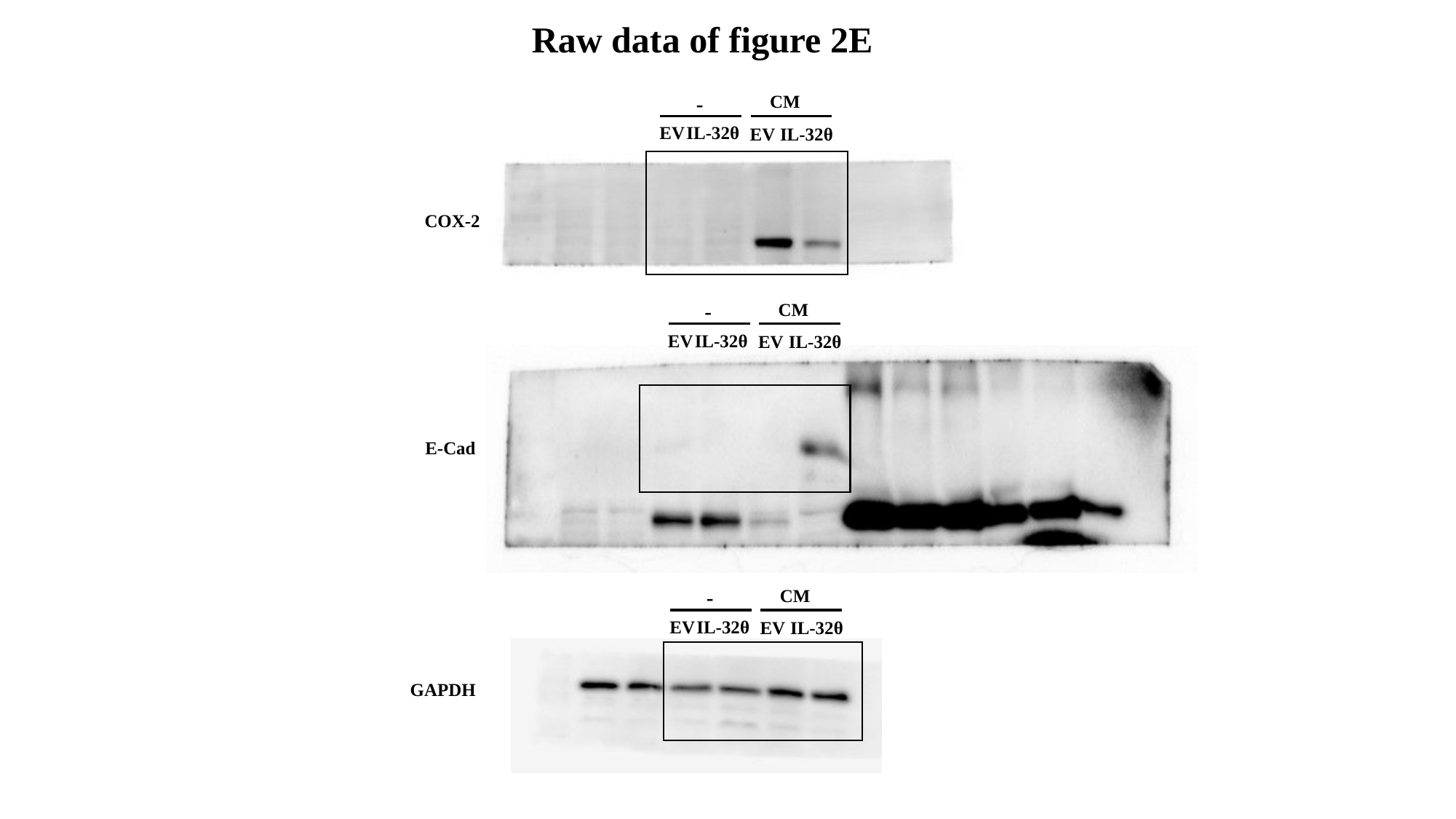

Raw data of figure 2E
-
CM
EV
IL-32θ
EV
IL-32θ
COX-2
-
CM
EV
IL-32θ
EV
IL-32θ
E-Cad
-
CM
EV
IL-32θ
EV
IL-32θ
GAPDH

## Slide 3
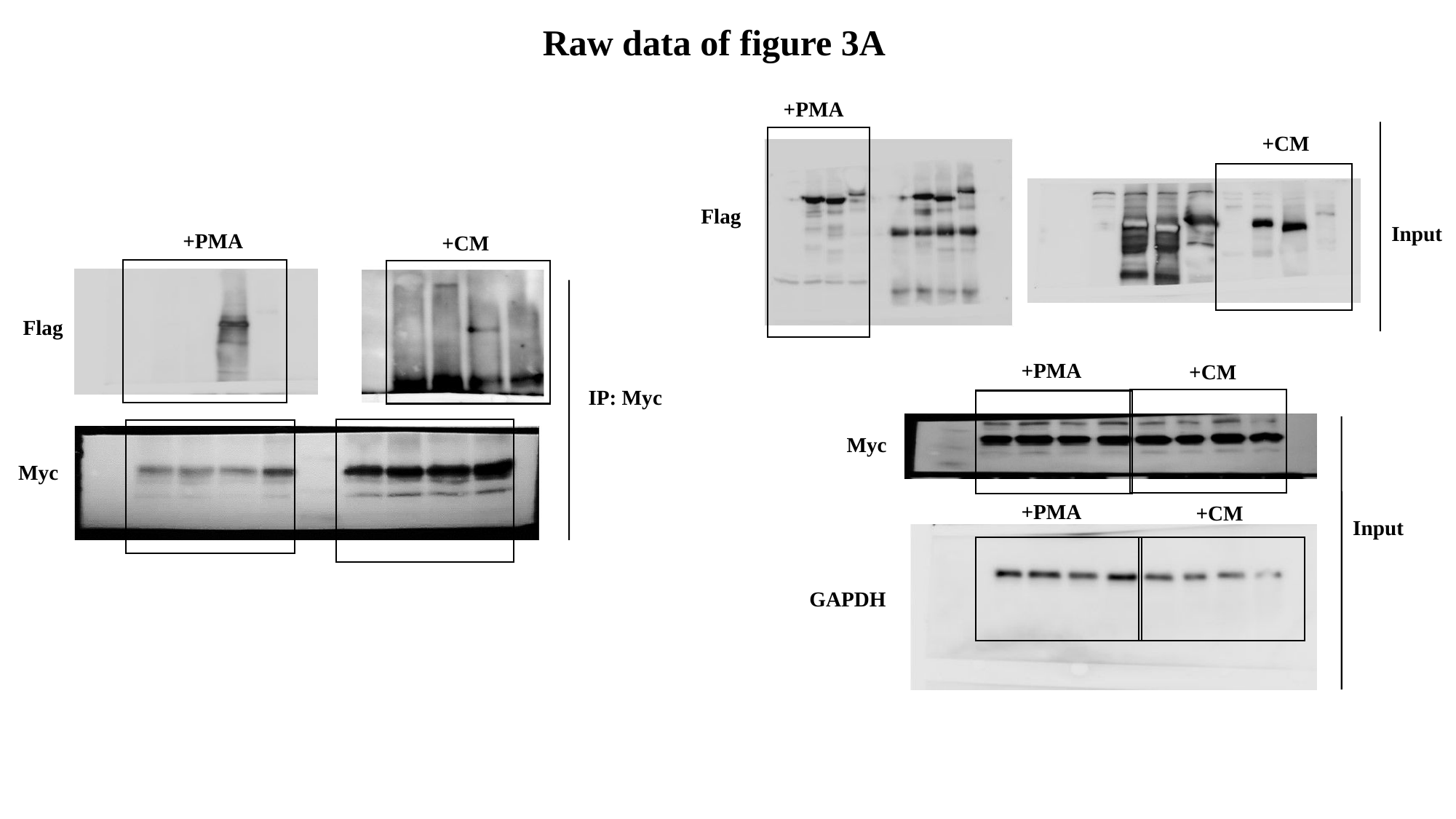

Raw data of figure 3A
+PMA
+CM
Flag
Input
+PMA
+CM
Flag
+PMA
+CM
IP: Myc
Myc
Myc
+PMA
+CM
Input
GAPDH

## Slide 4
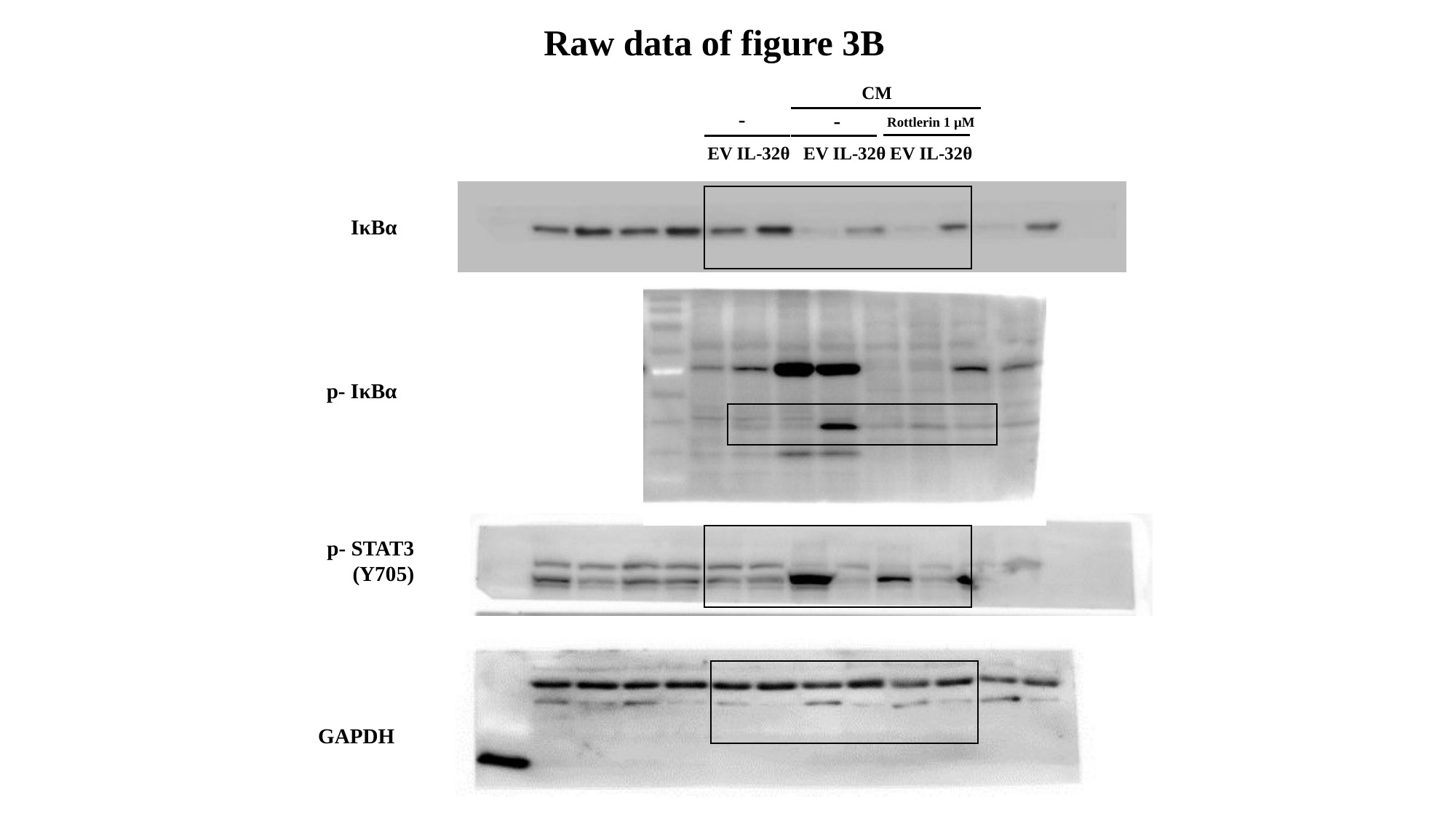

Raw data of figure 3B
CM
-
-
Rottlerin 1 µM
EV IL-32θ
EV IL-32θ
EV IL-32θ
IκBα
p- IκBα
p- STAT3 (Y705)
GAPDH

## Slide 5
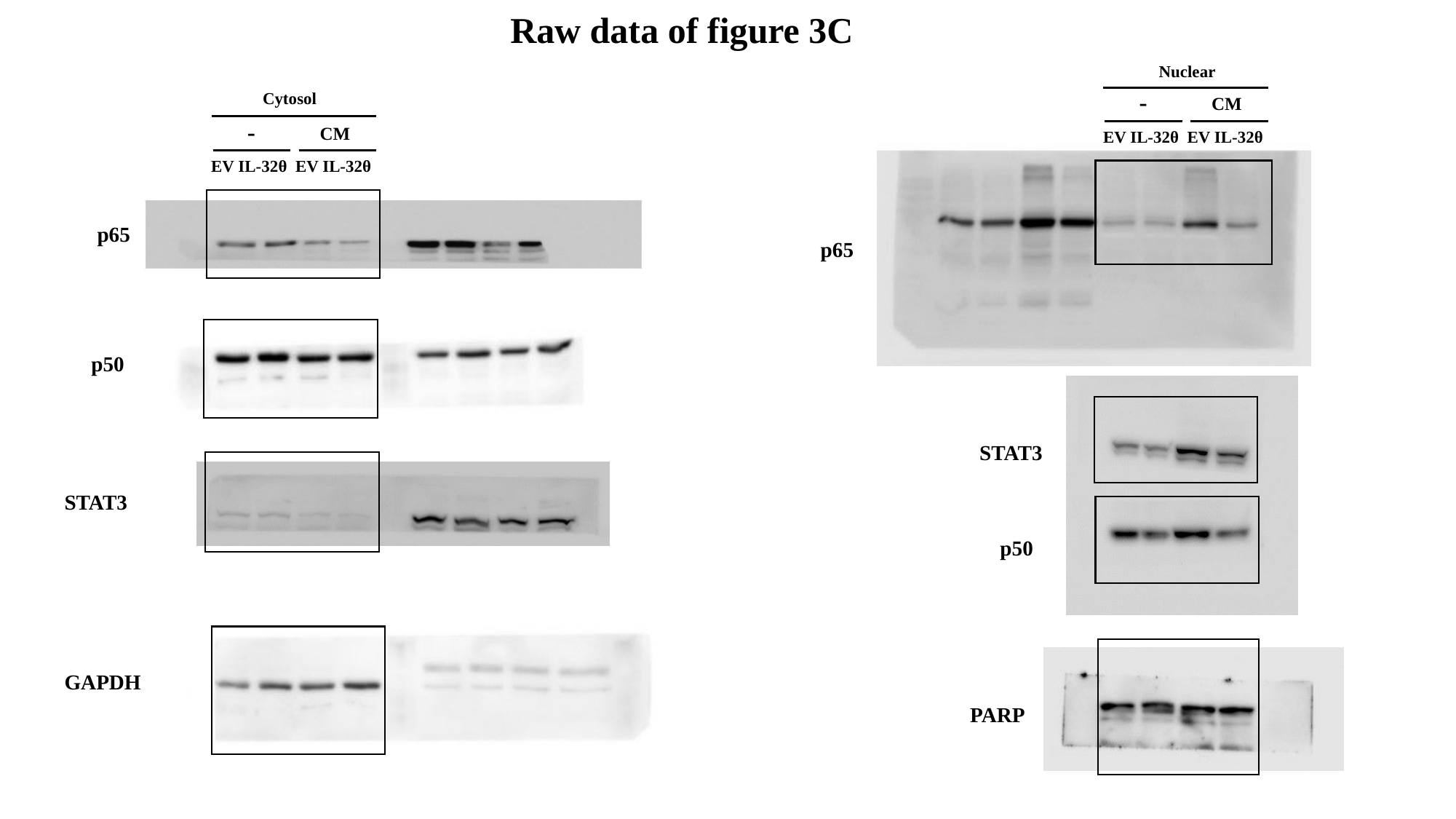

Raw data of figure 3C
Nuclear
-
Cytosol
CM
-
CM
EV IL-32θ
EV IL-32θ
EV IL-32θ
EV IL-32θ
p65
p65
p50
STAT3
STAT3
p50
GAPDH
PARP

## Slide 6
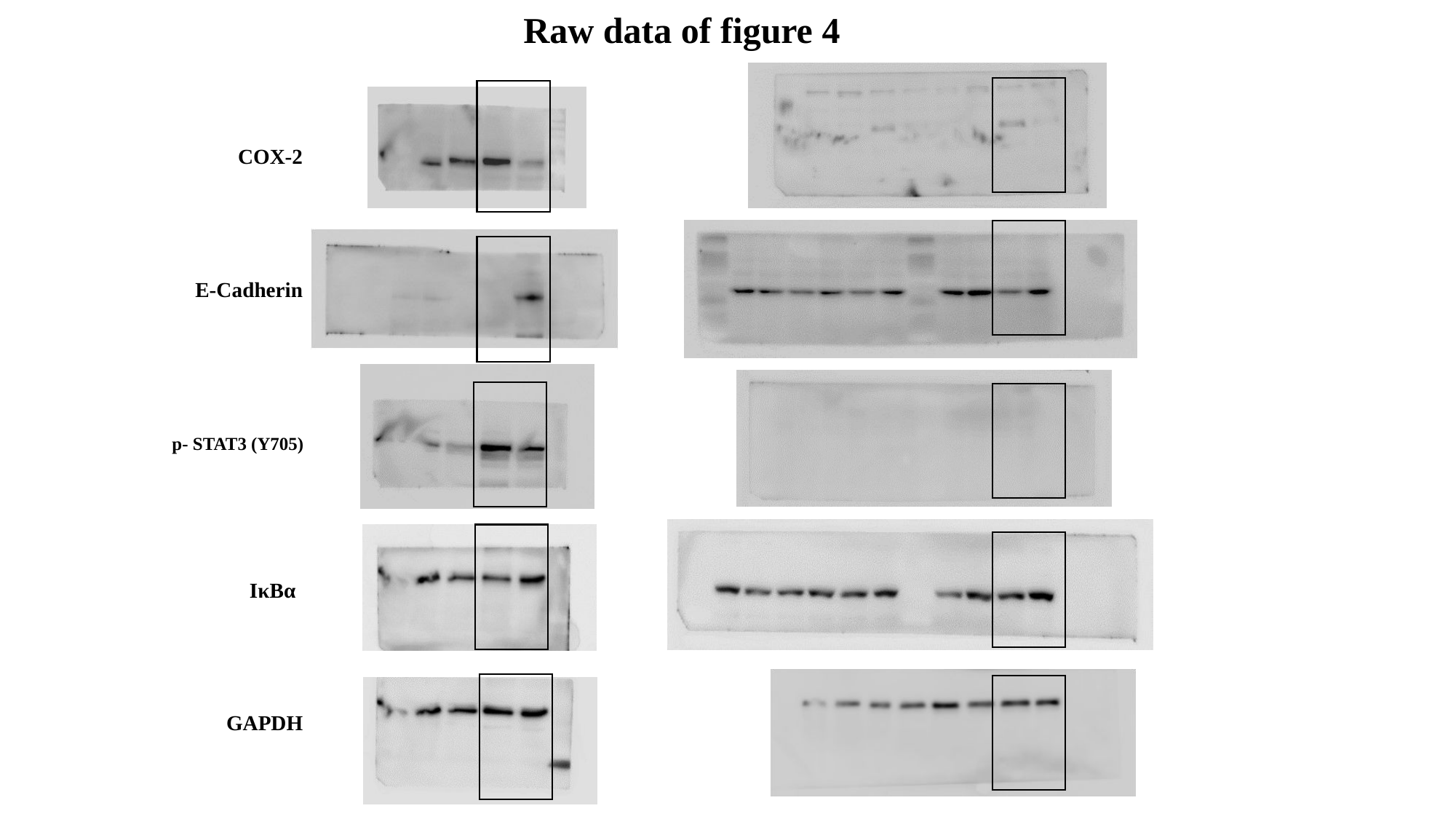

Raw data of figure 4
COX-2
E-Cadherin
p- STAT3 (Y705)
IκBα
GAPDH
